# The time dimension of neurodegeneration: the example of Friedreich’s ataxia

**Published:** 2017-02-20

**Authors:** Tommaso Vannocci, Annalisa Pastore

**Affiliations:** 1 Maurice Wohl Institute, King’s College London, 5 Cutcombe Rd., London SE5 9RT, UK; 2 Molecular Medicine Department, University of Pavia, Pavia, Italy

**Keywords:** Frataxin, NMR, Molecular complexes, Small angle X-ray scattering, Structural biology

An important theme which is quickly becoming a major aspect of modern Molecular Medicine involves the need to understand the time evolution of human diseases. It has become clear that when we enumerate the causes of a disease, it is often difficult to distinguish between causes, effects and co-existing phenomena. Do we have a headache because of a flu or we have a headache and also a flu? Is the pain in our joints due to inflammation or is the inflammation the consequence of another cause? Of course these are not new questions. However, the only thing researchers could do in the past was to sit back and speculate which could be the causal event. Now, for the first time, all is different. Modern genetics offers us new powerful tools which can allow us to control directly the causal progression of diseases rather than debating in the back scene. This possibility suddenly has added a temporal dimension to disease. An excellent example of what we are discussing is given by Freidreich’s Ataxia (FRDA), a recessive, autosomal disease with an incidence, in the Caucasian population, of 1 in 50,000 individuals^1^. FRDA is caused by the expansion of a GAA triplet in the first intron of the frataxin locus (FXN) which encodes a mitochondrial protein involved in the regulation of Fe-S cluster biogenesis^2^,^3^. This causes epigenetic modifications upstream of the gene, effectively silencing the expression of frataxin. Disease onset occurs usually before 25 years of age and roughly correlates inversely with the size of the GAA expansion^1^. Despite this overall rule, there is a huge disparity between patients’ phenotypes: expansions with similar size and large discernable variability in tissue damage suggests that, beside the primary mutations, genetic background, epigenetic and environmental factors largely modulate and direct phenotypes.

FRDA is characterized mainly by a progressive degeneration of large sensory neurons and by cardiomyopathies^4^. At a cellular level, the disease presents mitochondrial iron accumulation, increased oxidative stress and abnormalities in Fe-S cluster biogenesis^5^. Despite being extensively studied, it is still unclear what the initial cause of the disease and what secondary effects are (Figure 1A). It is difficult to create a temporal connection between the different phenotypes because patients’ samples, which are the most relevant samples to study, have been exposed to the condition for years since birth. To clarify the scene, a wide range of cellular and animal models have been created with the specific aim of establishing a clear initial time point from which FRDA phenotype can be induced and causes and secondary effects can be finally discriminated^6^. Although conceptually simple, creating a reliable, inducible model is far from easy and involves the difficult choice of the induction system (knockout vs knockdown) and of the type of organism/cell. Different models (yeast/mouse/human cells/drosophila) provide different results suggesting that association of different phenotypes does not necessarily imply a causal relationship.

Let’s discuss three examples of the conflicting evidence available, one involving an inducible knockout (KO) system based on mouse cells^7^, one based on drosophila^8^ and one on a yeast model^9^. All three studies followed the effects of FXN depletion in time, starting from a defined time point when FXN was repressed (Figure 1B). Due to the nature of the chosen cells/organism, progression was followed using different time scales (0-72 hours for the yeast model, 0-10 days for the mouse and 0-16 days for Drosophila cells) (Figure 2). Each study followed several biomarkers. We will focus on three specific different aspects to compare these studies: iron accumulation, oxidative stress and disruption of the Fe-S machinery.

The mouse model was a fibroblast cell line derived from transgenic mice. The endogenous FXN exon 4 was flanked by LoxP sites and the inducible KO promoted by a stably integrated Cre recombinase cassette under the control of a Tamoxifen dependent promoter^7^. The yeast model was the endogenous *YFH1* (homologue of FXN) gene under control of a TetO promoter (Tet-off system). The addition of tetracycline to the growth medium would inhibit expression of the gene^9^. The drosophila model presented instead a homozygous KO of the FXN-homologue fh gene only in photoreceptor neurons (mosaic mutant). The KO process was tissue specific and it is activated only during the development of the photoreceptors which was taken as the initial time point^8^ (Figure 3).

One of the initial observations in patients’ tissues was the presence of iron deposits^10^. Accordingly, iron overloading was detected as one of the first event to occur in both the yeast and the drosophila models, 14 h after *YFH1* repression with a 10-fold increase after 72 h^9^ and at day 3 (larval state) with marked neurodegeneration^8^. Surprisingly, the mouse model study identified iron accumulation at day 7 with substantial increase only at day 10, much after impairment of Fe-S-containing enzymes and Reactive Oxygen Species (ROS) production, a marker of oxidative stress^7^. Loss of the Fe-S enzyme aconitase activity (day 3) and ROS production (day 5) were the initial events triggered after Fxn KO. Moreno-Cermeno et al. concluded instead that reduction of aconitase activity and oxidative stress, measured by quantification of protein carbonylation at 24 h, were a direct effect of iron accumulation^9^. Increase in ROS production (monitored with the fluorescent dye dihydroethidium) was not detected, although Chen et al. could detect altered mitochondrial morphology already at day one in their drosophila model, indicating substantial mitochondrial dysfunction. Chen et al. did not monitor aconitase activity but managed to connect iron accumulation to an increase in sphingolipid synthesis (monitored by mass spectrometry) and subsequent dependent neurodegeneration spurring them to conclude that, at least in their model, ROS is not directly connected to neurodegeneration^8^.

These studies cleverly tried to differentiate between causes and effects by establishing a well-defined initial time for the development of the FRDA phenotype and closely following several different biomarkers during time but the results show both a temporal and causal discrepancy. This is especially true for the yeast model which seems to oppose completely the other two studies. Is thus ROS important or not for FRDA? Is there a way in which we could establish it?

In 2015, we exploited the CRISPR (Clustered Regularly-Interspaced Short Palindromic Repeats) system to engineer a cell line (based on Human Embryonic Kidney cells HEK293) where the presence of an exogenous, inducible FXN (iFXN) gene rescues the cells from the biallelic knockout of the endogenous FXN genes^11^. Our aim was to generate a cell line that could be used to provide a temporal relationship of the disease events to be able to establish conclusively the molecular mechanisms that trigger FRDA and distinguish them from the secondary effects. We used the immortalized HEK293 (Human Embryonic Kidney) cell line. Although this line is not directly relevant to the affected tissues of FRDA patients, it was a convenient choice to establish the proof of principle for the approach in the first instance.

The choice of the specific CRISPR used was dictated by the possibility to have the proximity of its target sequence to exon 4 of FXN. We created in this way a targeting construct (pFSVpur-LoxP-TC-I4) that, when integrated by homologous recombination, was able to excise the exon completely and replaced it with a puromycin resistance cassette. We then produced knockout of both FXN alleles which required two rounds of transfection with CRISPR-I4 and the targeting construct because simultaneous homozygous FXN knockout is a rare event. The presence of the puromycin cassette flanked by two Lox-P sites allowed us to select the targeted cells in the first round followed by Cre recombinase-mediated excision of the puromycin cassette and a second round of targeting using the same pFSVpur-LoxP-TC-I4 construct (Figure 3). The targeting experiments carried out with CRISPR-I4 and pFSVpur-LoxP-TC-I4 showed a targeting frequency of ~50% to be compared to a 0% frequency when cells were transfected with only pFSVpur-LoxP-TC-I4 targeting construct. This step therefore proved the feasibility of successfully performing gene editing at the *FXN* locus. The inducible iFXN cassette allowed us to modulate the amount of frataxin in the cell by over or under–expression of the gene itself.

The successful use of this system established an important step forward which will allow us to follow and understand the early stages of FRDA development and progression. The system can be used now to monitor the effects of FXN depletion with biomarkers that detect cellular ROS, and with indicators of iron-sulfur cluster formation such as aconitase levels which has successfully been used in FRDA studies^12^. The approach developed will not only give us an insight in the disease mechanism (under-expression) but could also provide useful information on the effects of FXN concentration on several mitochondrial pathways (over-expression).

The next logical step will require the development of a new cellular model that mimics more faithfully the tissues affected by FRDA. To approach this problem, we are planning to use the genetic engineering tools that we recently developed to obtain an inducible system based on inducible Pluripotent Stem (iPS) cells. iPS cells have the advantage of differentiating in FRDA-relevant cell types like sensory neurons and cardiomyocytes and have also the advantage of having a normal karyotype (normal diploid, normal XY). A word of caution may however be spent to highlight that, working on cultured cells (including iPS), which largely use culture medium as an endless spillway and grow in peculiar conditions, could in principle influence the results and lead to incorrect conclusions. Additionally, clonal cells increase the reliability and consistency between experiments thanks to the lack of genotype variation. However, this lack of variability could also create biases in the conclusions by limiting the possible effects produced by different genetic and epigenetic backgrounds. Particular care will thus needed to be paid to ensure that any conclusion from this otherwise promising model system may faithfully represent a close approximation to reality.

We hope that in this way we will contribute to a new dimension of disease comprehension: time. We can easily predict that the concept of following disease progression will become increasingly important in future years.

## Figures and Tables

**Figure 1:**
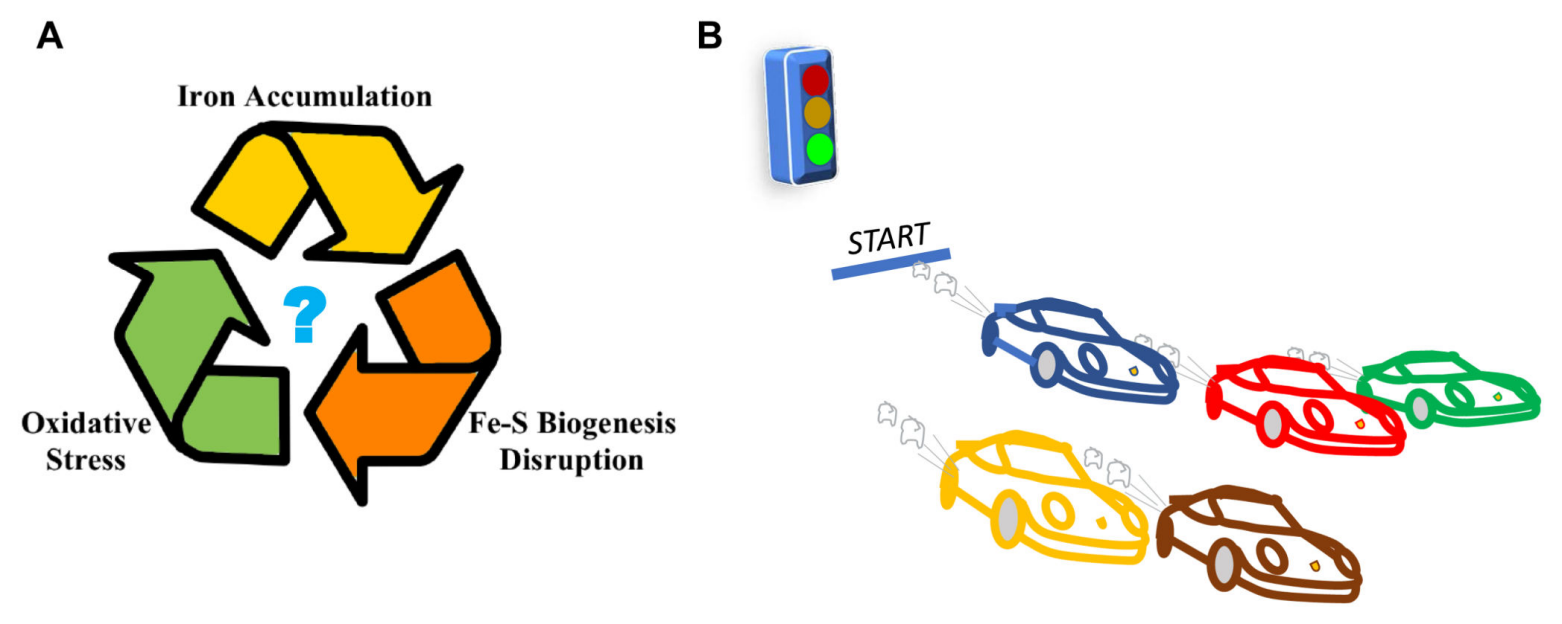
The importance of establishing a causal progression of FRDA. A) Fuzzy relationship between observed phenotypes. It is difficult to distinguish the causes from the effects; B) The best way to follow progression is when we can control the events from a well-defined starting point, as in car race.

**Figure 2:**
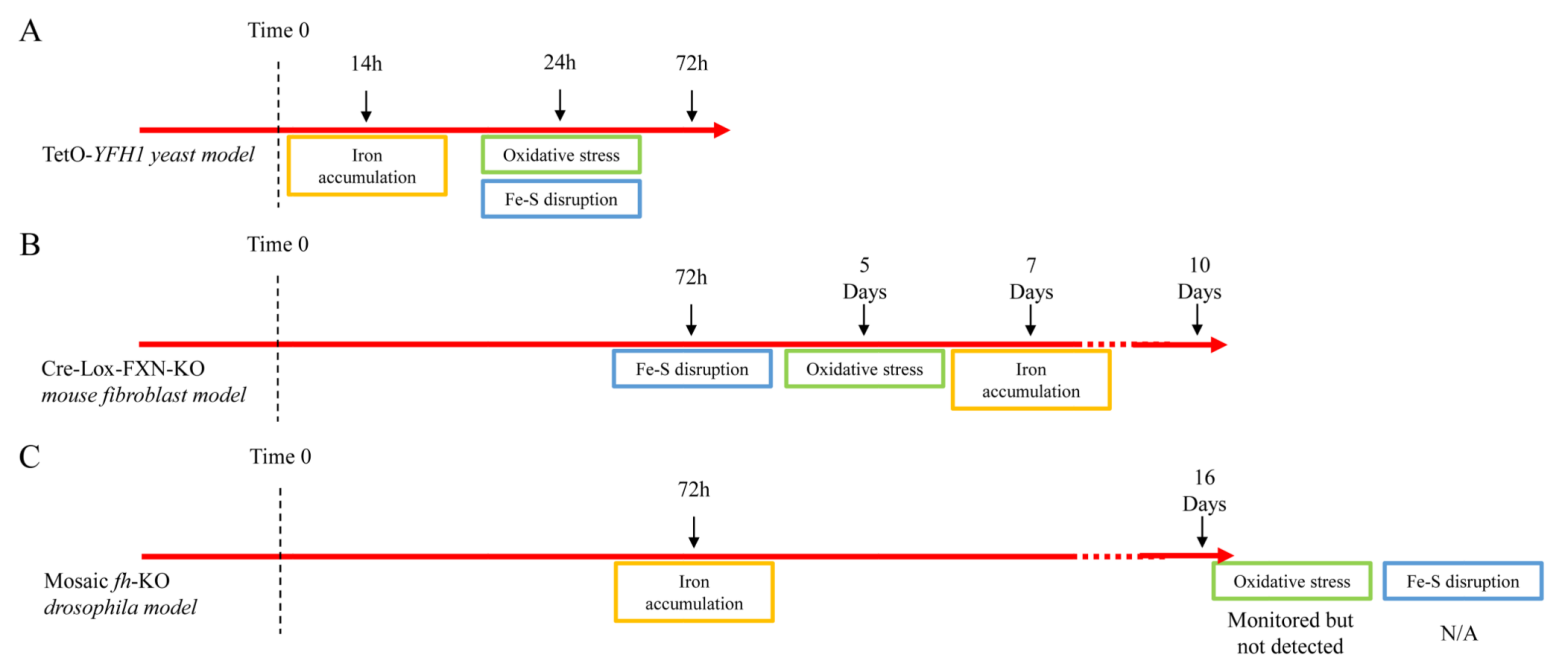
Summary of the outcomes found in the three different studies shown against a horizontal time. A) TetO-YFH1 yeast model^9^; B) Cre-Lox-FXN-KO mouse fibroblast model^7^; C) Mosaic *fh-*KO drosophila model^8^.

**Figure 3:**
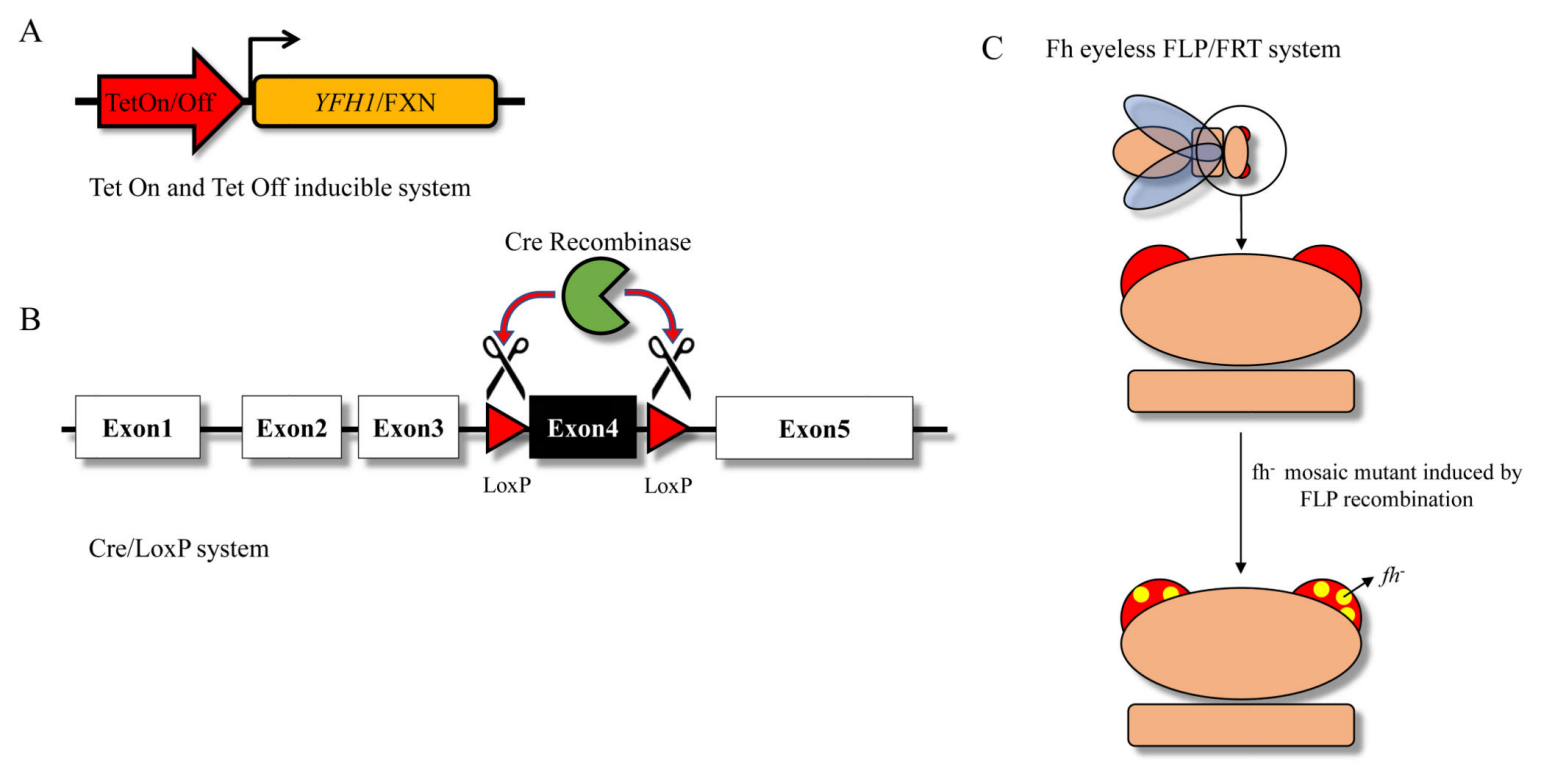
Representation of the different frataxin KO inducible systems described in this review. A) Knockout of the endogenous frataxin gene is rescued by the presence of an exogenous, inducible frataxin cassette under the control of the Tet-On^11^ or Tet-Off system^9^; B) Knockout of the mouse FXN gene is mediated by the tamoxifen dependent expression of Cre recombinase that promote the excision of the LoxP-flanked exon 4 of the gene^7^; C) Drosophila mosaic mutant: homozygous KO of the FXN-homologue fh gene is present only in photoreceptor neurons^8^.
